# Integrative Transcriptomics, Machine Learning, and Molecular Dynamics Reveal Honghua Longdan (*Gentiana rhodantha*)‑Modulated Therapeutic Targets in Bladder Cancer

**DOI:** 10.1021/acsomega.5c12612

**Published:** 2026-03-06

**Authors:** Qinsha Wang, Haihong Wang, Peng Lan, Bing Yang, Jia Deng, Kangmin Zhou, Dongxin Tang

**Affiliations:** † 326770 Guizhou University of Traditional Chinese Medicine, No. 4, Dongqing Road, Huaxi District, Guiyang 550002, Guizhou Province, China; ‡ Department of Emergency Medicine, The Traditional Chinese Medicine Hospital of Longquanyi, 222 Qingtaishan Road, Damian Subdistrict, Chengdu 610100, Sichuan Province, China

## Abstract

Bladder cancer (BC) has a high recurrence rate and marked molecular heterogeneity, yet effective biomarkers and druggable targets remain scarce. Honghua Longdan (*Gentiana rhodantha* Franch., GR), a traditional Chinese medicine, exhibits antitumor activity, but its therapeutic targets and mechanisms in BC remain poorly defined. Here, differentially expressed genes (DEGs) in BC from TCGA-BLCA and GEO were integrated with weighted gene coexpression network analysis (WGCNA) to identify BC-related gene modules. Active GR-derived compounds were obtained from HERB v 2.0 and SymMap. Three machine-learning algorithms were employed to identify hub genes and build a neural-network diagnostic model. Functional enrichment, survival, and gene set enrichment analyses together with immunohistochemistry (IHC) were used to characterize key genes. Molecular docking and all-atom molecular dynamics simulations were performed to validate interactions between hub proteins and GR-derived compounds. Integrated analyses identified eight core drug–disease genes enriched in cell-cycle-related pathways. Six hub genes were consistently selected by the three algorithms, and the neural-network model showed excellent diagnostic performance. High CCNB1 expression was significantly associated with poorer overall survival (OS), and its expression is markedly upregulated in BC tissues. In silico simulations suggested that the GR-derived iridoid glycoside swertiamarin forms a stable complex with CCNB1. This study integrates network pharmacology, machine learning, and computational biophysics to identify CCNB1 as a biomarker with diagnostic and prognostic value in BC. The predicted interaction between swertiamarin and CCNB1 suggests that CCNB1 is a GR-mediated therapeutic target and supports the rational discovery of traditional Chinese medicine-derived therapeutics.

## Graphical abstract

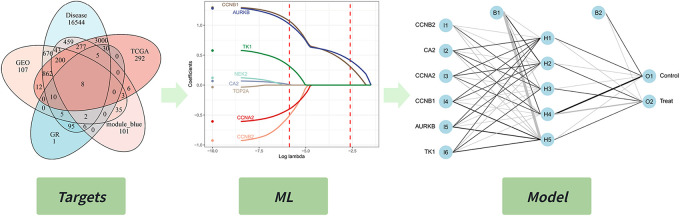

## Introduction

Bladder cancer (BC) is one of the most common malignancies of the urinary tract worldwide and is characterized by frequent recurrence, pronounced heterogeneity, and high invasiveness, posing a major threat to human health.^1^ According to the latest GLOBOCAN 2020 estimates, approximately 570,000 new BC cases and more than 210,000 BC-related deaths occurred worldwide in 2020, and these numbers are projected to increase by ∼73 and ∼87%, respectively, by 2040.^2–5^ Despite advances in multimodal treatments, including surgery, chemotherapy, radiotherapy, and immunotherapy, the 5-year overall survival of patients with muscle-invasive BC (MIBC) remains at ∼50% and has improved only marginally over recent decades.^6^ Current imaging techniques and urine cytology lack sufficient sensitivity and specificity for early detection and surveillance of recurrence, underscoring the urgent need for reliable molecular biomarkers and therapeutic targets to enable early screening, risk stratification, and precision treatment of BC.^7^

In recent years, natural products have attracted increasing interest in anticancer research because of their structural diversity, favorable biocompatibility, and unique capacity for multitarget regulation.^8^ According to the 2020 edition of the Chinese Pharmacopoeia,^9^ GR clears heat and detoxifies, removes dampness and relieves jaundice, and is commonly prescribed for damp-heat jaundice, dysuria, and lung-heat cough.^10^ It is also widely used by several ethnic minority groups in southwestern China, including the Bai, Dong, Lisu, and Miao peoples. Modern pharmacological studies indicate that its major active constituents, including swertiamarin, gentiopicroside, and quercetin, possess anti-inflammatory,^11,12^ antioxidant, hepatoprotective,^13^ and antitumor activities in diverse disease models.^14,15^ GR is widely used to treat urinary system disorders; however, its mechanisms of action, key targets, and molecular basis in BC have not been systematically elucidated, which limits its further clinical development.

Network pharmacology provides a powerful framework for elucidating the complex characteristics of traditional Chinese medicines with multiple components, targets, and pathways,^16^ but its predictions still require validation using systems-biology approaches. Machine-learning-based integrative multiomics analyses offer efficient tools for screening key cancer-related genes and have shown excellent performance in identifying disease-driving molecules.^17^ In addition, molecular docking and molecular dynamics simulations are widely used to validate drug–target interactions and to predict potential antitumor mechanisms.^18^ Integrating network pharmacology, machine learning, and computer-aided simulations is expected to establish a continuous evidence chain from macroscopic big-data prediction to microscopic structural validation, thereby improving both the reliability and novelty of drug-target identification.

Bulk transcriptomic data from multiple TCGA and GEO cohorts were integrated, and machine-learning algorithms were applied to identify core BC-related targets and construct a multigene neural-network diagnostic model. Subsequently, molecular docking and molecular dynamics simulations were performed to characterize the interactions between key GR constituents and target proteins, thereby elucidating their potential antitumor mechanisms. This work provides potential biomarkers for the early diagnosis of BC and offers a theoretical basis for the development of new targeted therapeutics. The overall workflow of this study is summarized in Figure 1.

**Figure 1:**
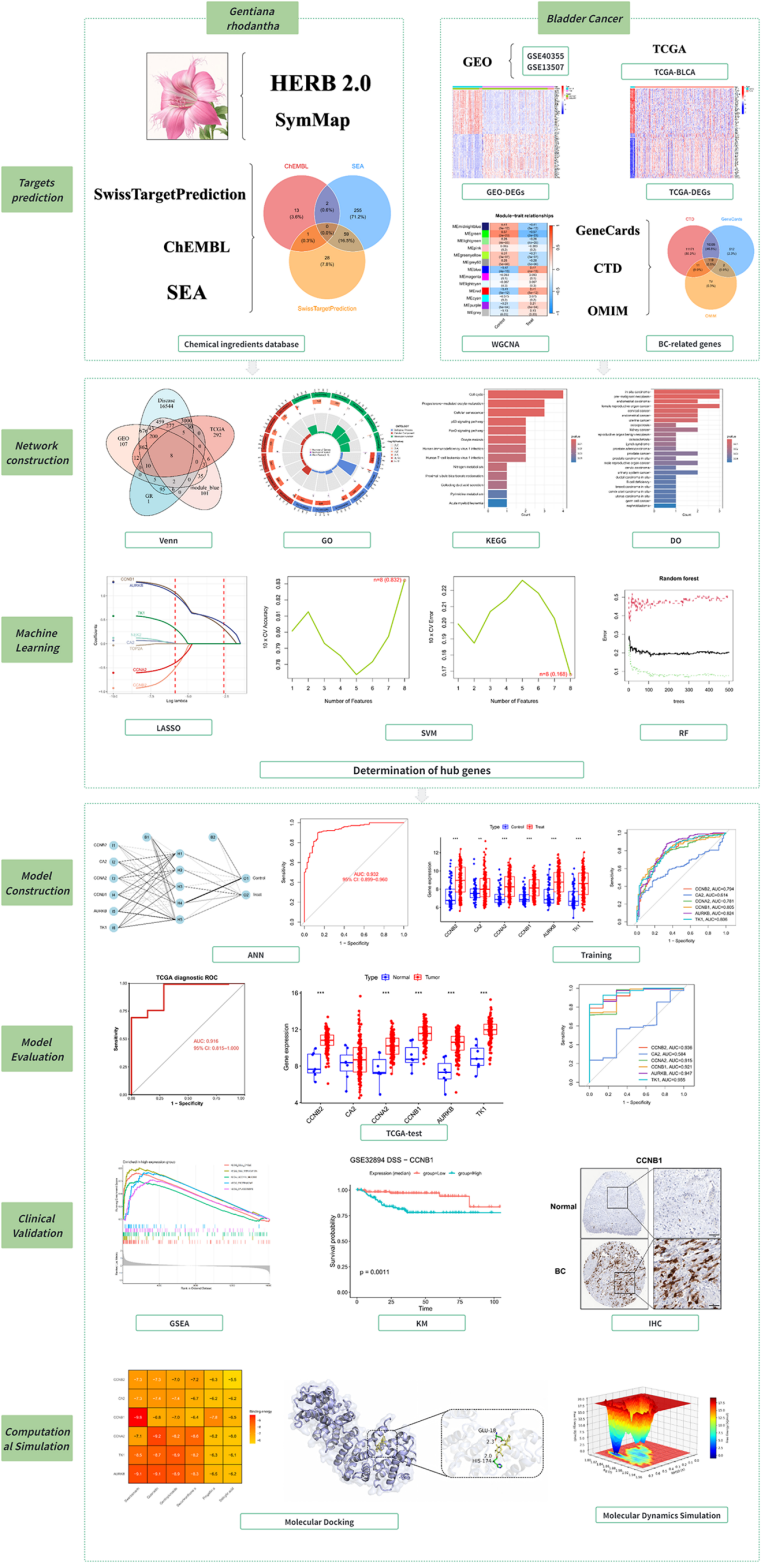
Overall workflow of the study (schematic illustration of *Gentiana rhodantha* created by the authors).

## Results

### BC-Related Disease Genes

In the TCGA-BLCA cohort, differential expression analysis between 19 normal and 412 tumor samples identified 4704 TCGA-derived differentially expressed genes (DEGs) under the thresholds of adjusted *P* < 0.05 and |log2FC| > 1, including 2700 upregulated and 2004 downregulated genes in tumor versus normal tissues (Figures 2A and S1A). To improve the consistency of external cohorts, GEO data sets GSE13507 (Control = 67, Treat = 165) and GSE40355 (Control = 8, Treat = 16) were merged, resulting in a batch-corrected cohort comprising 75 Control and 181 Treat samples, with batch effects effectively mitigated (Figures 2B,C and S1C). Differential expression analysis of the merged GEO cohort identified 1962 GEO-derived DEGs using |log2FC| > 0.585 and adjusted *P* < 0.05, including 641 upregulated and 1321 downregulated genes (Figures 2B and S1B and Table S1). In addition, a weighted gene coexpression network was constructed using WGCNA, and β = 5 was selected as the soft-thresholding power for subsequent analyses. Module–trait correlation analysis indicated that the blue module was most strongly and negatively correlated with the Treat status (*r* = −0.47, *P* = 4 × 10^–15^), and genes in this module showed high gene significance (cor = 0.71, *P* = 2.7 × 10^–176^) (Figure 2D,F), suggesting that the blue module may be enriched for key genes closely associated with BC progression­(Figure S1D,E). In total, 22,043 potential BC-related genes were integrated from the CTD, GeneCards, and OMIM databases (Figure 2H), providing candidate genes for subsequent drug–disease intersection analyses.

**Figure 2:**
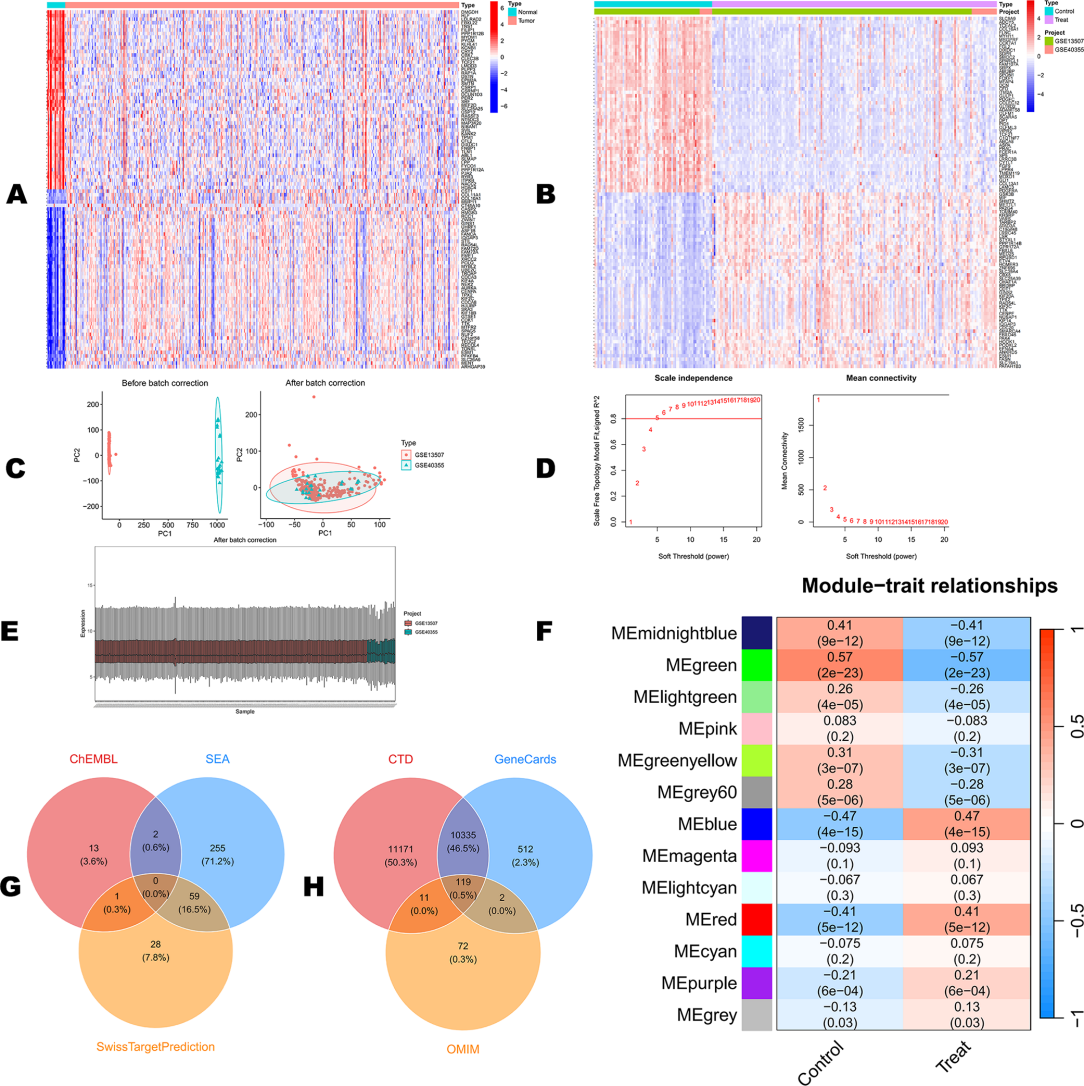
Identification of bladder cancer-related genes from TCGA, GEO data sets, WGCNA analysis, and disease databases. (A) Heatmap of differentially expressed genes (DEGs) in the TCGA cohort (tumor vs normal). (B) Heatmap of the merged GEO data sets (GSE13507 and GSE40355); the top annotation indicates group (normal/tumor) and data set source. (C) Principal component analysis (PCA) before and after batch-effect correction to evaluate batch removal. (D) Selection of the soft-thresholding power (β) for WGCNA based on scale independence and mean connectivity. (E) Box plots of expression distributions across samples after batch correction to assess overall comparability. (F) Module–trait relationship heatmap showing correlations between module eigengenes and the Normal/Tumor groups. (G) Venn diagram of predicted GR-associated targets intersecting among ChEMBL, SEA, and SwissTargetPrediction. (H) Venn diagram of bladder cancer-associated genes intersecting among CTD, GeneCards, and OMIM.

### Target Prediction for GR

Based on the HERB 2.0 and SymMap databases, we ultimately identified six representative active constituents of GR (Table 1). Target prediction showed that SEA yielded 316 candidate targets, of which 255 were unique to SEA; SwissTargetPrediction identified 88 targets, including 28 unique targets; and ChEMBL provided 16 targets, 13 of which were specific to this platform (Figure 2G). Although partial overlap was observed among the three platforms, each also contained a set of platform-specific targets, indicating that combining multiple platforms increases the coverage and reliability of the target prediction.

**Table 1:** Core Compounds of Screened GR

ingredient id	ingredient name	ingredient alias name	molecular formula	canonical smiles
HBIN027510	gentiopicroside	Gentiopicrin; 20831-76-9; UNII-0WE09Z21RC; 0WE09Z21RC; CHEBI:5321; DTXSID40878043; EINECS 244-070-2; NSC 606402; NSC-606402	C16H20O9	C=CC1C­(OC=C2C1=CCOC2=O)­OC3C­(C(C(C(O3)­CO)O)O)O
HBIN040797	Progallin a	ETHYL GALLATE; 831-61-8; ethyl 3,4,5-trihydroxybenzoate; gallic acid ethyl ester; Phyllemblin; Ethylgallate; Nipagallin A; Nipa No. 48; gallic acid, ethyl ester	C9H10O5	CCOC­(=O)­C1=CC­(=C(C­(=C1)­O)O)O
HBIN041495	quercetin	117-39-5; Sophoretin; Meletin; Quercetine; Xanthaurine; Quercetol; Quertine; Quercitin; 2-(3,4-dihydroxyphenyl)-3,5,7-trihydroxy-4H-chromen-4-one	C15H10O7	C1=CC­(=C(C=C1C2=C­(C(=O)­C3=C­(C=C­(C=C3O2)­O)O)O)O)O
HBIN042852	salicylic acid	2-hydroxybenzoic acid; 69-72-7; *o*-hydroxybenzoic acid; 2-carboxyphenol; *o*-carboxyphenol; Rutranex; Salonil; Retarder W; Duoplant	C7H6O3	C1=CC=C­(C(=C1)­C(=O)­O)O
HBIN043601	Securixanthone a	CHEMBL5287044; 1,3,7-trihydroxy-2,8-dimethoxyxanthone; BDBM50611921; 1,3,7-trihydroxy-2,8-dimethoxy-xanthen-9-one; 1,3,7-trihydroxy-2,8-dimethoxy-9*H*-xanthen-9-one; 9*H*-xanthen-9-one, 1,3,7-trihydroxy-2,8-dimethoxy-; InChI = 1/C15H12O7/c1–20–14–6(16)3–4–8–11(14)12(18)10–9(22–8)5–7(17)15(21–2)13(10)19/h3–5,16–17,19H,1–2H	C15H12O7	COC1=C­(C=CC2=C1C­(=O)­C3=C­(O2)­C=C(C­(=C3O)­OC)O)O
HBIN045185	swertiamarin	17388-39-5; Swertiamarine; Swertiamaroside; SWERTAMARIN; UNII-4038595T7Y; MFCD07783984; BRN 0055278; Swertimarine; MLS002473253	C16H22O10	C=CC1C­(OC=C2C1­(CCOC2=O)­O)­OC3C­(C(C(C­(O3)­CO)O)O)O

### Disease–Drug Intersection Analysis Identifies Eight Core Genes Involved in Cell-Cycle Regulation

To identify key genes linked to both BC pathogenesis and the active constituents of GR, we intersected GEO–DEGs, TCGA-DEGs, disease-associated genes (DAGs), GR target genes (DTGs), and genes from the WGCNA blue coexpression module (module_blue), and identified eight shared drug–disease core genes (Figure 3A). GO enrichment analysis indicated that these eight core genes are mainly involved in biological processes, such as cell-cycle regulation, chromosome segregation, and protein kinase activity (Figure 3B–D). KEGG pathway analysis further showed that they are significantly enriched in signaling pathways related to cell-cycle progression and cellular stress responses (Figure 3E,F), suggesting that these genes play crucial roles in key events such as the G2/M transition, mitosis, and DNA replication. DO enrichment analysis revealed that these core genes are significantly enriched in multiple cancer-related diseases, particularly urinary system cancers, where they showed high GeneRatios and strong statistical significance (Figure 3G,H), further supporting their close association with the occurrence and progression of urinary tract malignancies.

**Figure 3:**
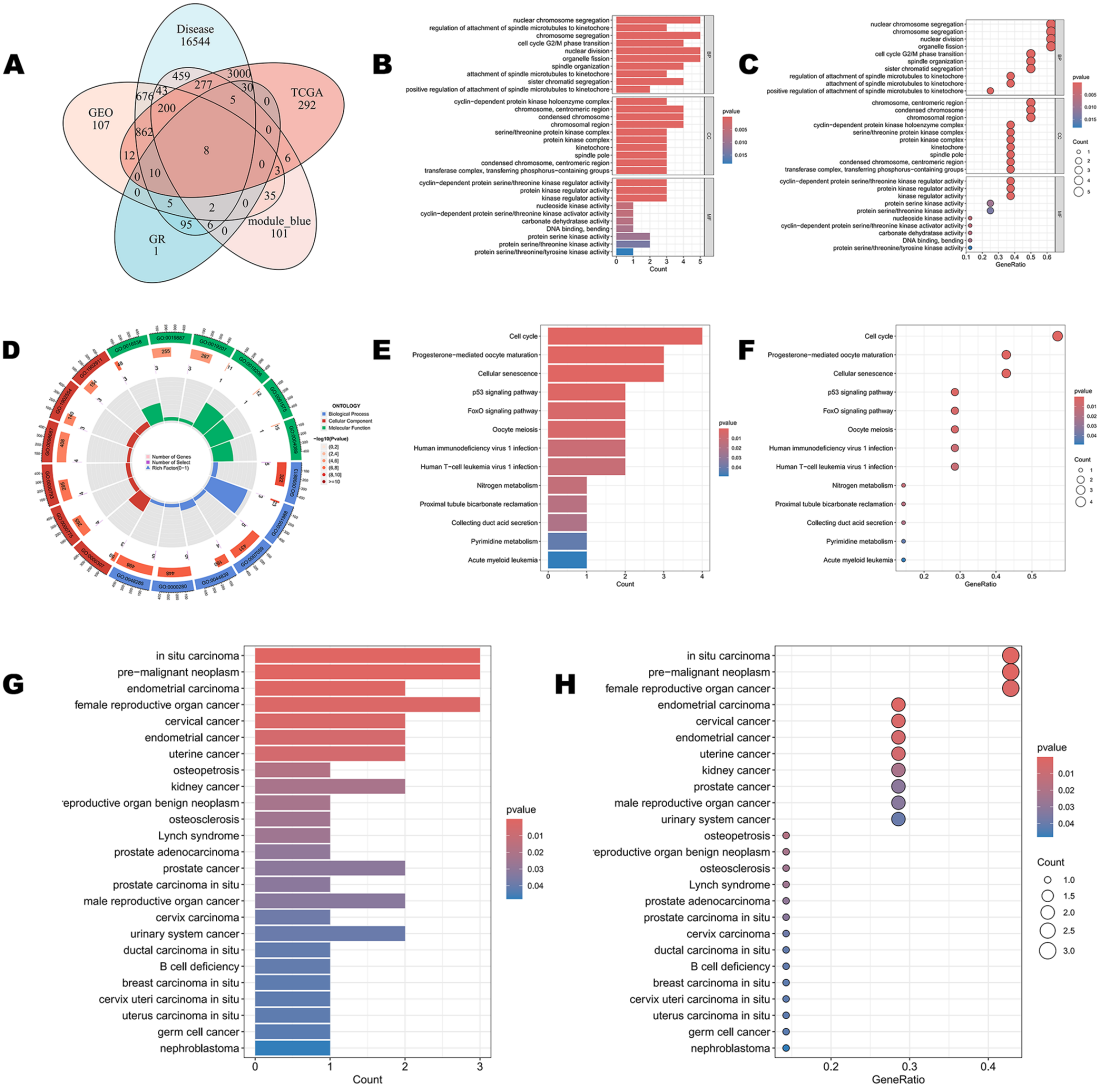
Identification of key genes and pathways in bladder cancer. (A) Venn diagram showing the intersection of genes from five gene sets: Disease (DAGs), GEO (GEO–DEGs), TCGA (TCGA-DEGs), GR (DTGs), and module_blue (WGCNA blue module). (B) Bar plot of GO (Gene Ontology) enrichment analysis results. (C) Dot plot of GO (Gene Ontology) enrichment analysis results. The size of the dot represents the gene count, and the color represents the *p*-value. (D) Circos plot of GO (Gene Ontology) enrichment analysis results. (E) Bar plot of KEGG (Kyoto Encyclopedia of Genes and Genomes) pathway enrichment analysis results. (F) Dot plot of KEGG (Kyoto Encyclopedia of Genes and Genomes) pathway enrichment analysis results. The size of the dot represents the gene count, and the color represents the *p*-value. (G) Bar plot of DO (Disease Ontology) enrichment analysis results. (H) Dot plot of DO (Disease Ontology) enrichment analysis results. The size of the dot represents the gene count, and the color represents the *p*-value.

### Identification of Core Genes by Machine Learning

Integrating the outputs of the three machine-learning algorithms, six genes, CA2, AURKB, CCNB1, CCNB2, CCNA2, and TK1 were simultaneously selected by LASSO, SVM-RFE, and random forest (RF) (Figure 4A–G). Protein–protein interaction network analysis further showed that these six genes occupy central positions within the network, and GeneMANIA analysis (Figure 4H) demonstrated that this core gene set is strongly correlated with canonical cell-cycle regulators, including the cyclin-dependent kinase CDK1 and cyclins CCNE1 and CCNE2, as well as factors such as CDC20, BIRC5, and NCAPH that participate in mitotic spindle assembly, chromosome condensation, and cytokinesis. These proteins act cooperatively in processes such as the G2/M transition, mitotic progression, and S-phase DNA synthesis, suggesting that they exert hub regulatory functions in the initiation and progression of BC. Consequently, they were defined as core hub genes in this study and were used for subsequent diagnostic model construction and functional analyses.

**Figure 4:**
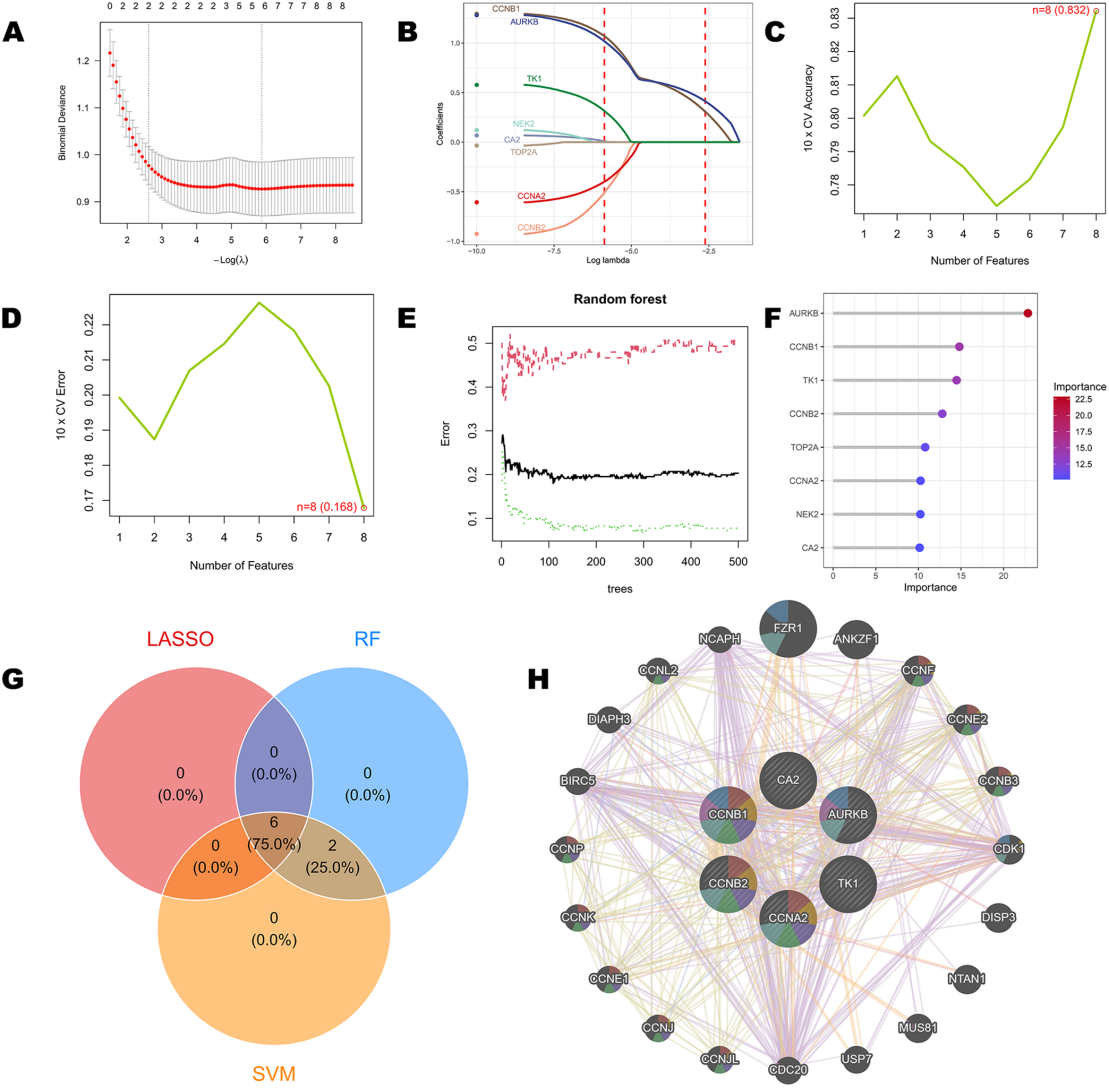
Identification of key hub genes using machine learning algorithms and GeneMANIA network analysis. (A) LASSO regression analysis for feature selection. (B) LASSO coefficient profiles of the selected features. (C) SVM cross-validation accuracy plot vs number of features. (D) SVM cross-validation error plot vs number of features. (E) Random Forest (RF) error rate vs number of trees. (F) Feature importance ranking from RF. (G) Venn diagram showing the overlap of key genes selected by LASSO, RF, and SVM. (H) GeneMANIA network analysis of the identified key genes. Node size is proportional to the number of interactions. Edge colors represent different types of interactions.

### Hub Gene-Based Neural-Network Diagnostic Model Demonstrates High Diagnostic Accuracy in Bladder Cancer

Box plots revealed that all six hub genes were significantly upregulated in tumor tissues in the training set (Figure 5A), suggesting their involvement in BC initiation and progression. A three-layer neural-network diagnostic model was constructed using the expression levels of the six hub genes as input features (Figure 5B). The contributions of individual genes to the classification of “tumor” versus “normal” varied, with CCNB1, AURKB, and TK1 exhibiting relatively higher weights. The area under the ROC curve (AUC) for the model was 0.932 (95% CI: 0.899–0.960), indicating high overall diagnostic accuracy (Figure 5C). Single-gene ROC analysis showed that AURKB and CCNB1 had comparatively stronger diagnostic powers (Figure 5D). In the TCGA testing set, the model achieved an AUC of 0.916 (Figure 5E), further supporting its robust discriminatory ability. The expression patterns of the five hub genes with significant upregulation in tumor tissues (CCNB2, CCNB1, AURKB, and TK1) in the testing set were consistent with those in the training set (Figure 5F,G), supporting their stability as diagnostic biomarkers. However, CA2 exhibited a consistent direction of change but did not reach statistical significance in the testing cohort (Figure 5G), suggesting the gene’s limited role as a potential biomarker for BC diagnosis.

**Figure 5:**
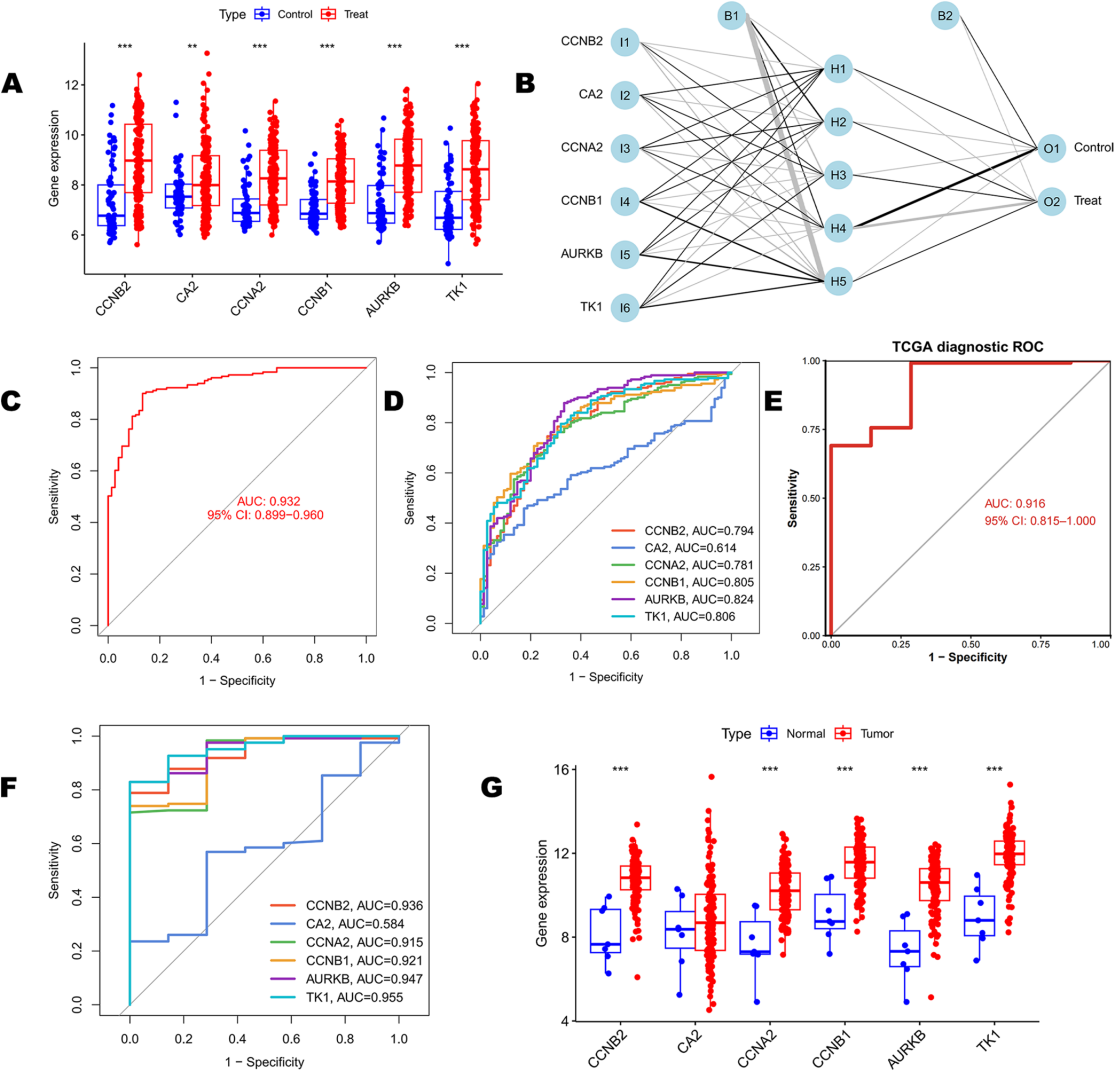
Integrated analysis of core gene diagnostic performance using an artificial neural-network model. (A) Differential expression of six core genes between tumor vs normal tissues (*t*-test; **P < 0.01, ***P < 0.001). (B) Architecture of the artificial neural network (ANN) with one input layer (I1–I6, corresponding to six genes), one hidden layer (H1–H5), and one output layer (O1–O2 for binary classification). (C) Receiver operating characteristic (ROC) curve of the ANN model on the training set (AUC = 0.93). (D) Single-gene ROC analysis of core genes in the training set. (E) ROC curve validating ANN performance on the independent TCGA test cohort (AUC = 0.92). (F) Validation of single-gene diagnostic efficacy in the TCGA cohort. (G) Expression validation of core genes in TCGA tumor vs normal tissues (**P < 0.01, ***P < 0.001).

### High CCNB1 Expression Is Associated with Poor Prognosis in Patients with BC

Kaplan–Meier survival analyses showed that among the six hub genes, elevated CA2 and CCNB1 expression was significantly correlated with shorter overall survival (OS) (Figures 6A and S2A). In two independent GEO cohorts, disease-specific survival (DSS) analysis in GSE32894 and overall survival (OS) analysis in GSE13507 further demonstrated that, except for CA2, high expression of CCNB1, AURKB, CCNA2, CCNB2, and TK1 was significantly associated with unfavorable survival outcomes (Figures 6B,C and S2B,C). Given the consistent prognostic association of CCNB1 across the TCGA cohort and the two GEO validation cohorts, CCNB1 was selected for subsequent in-depth analyses.

**Figure 6:**
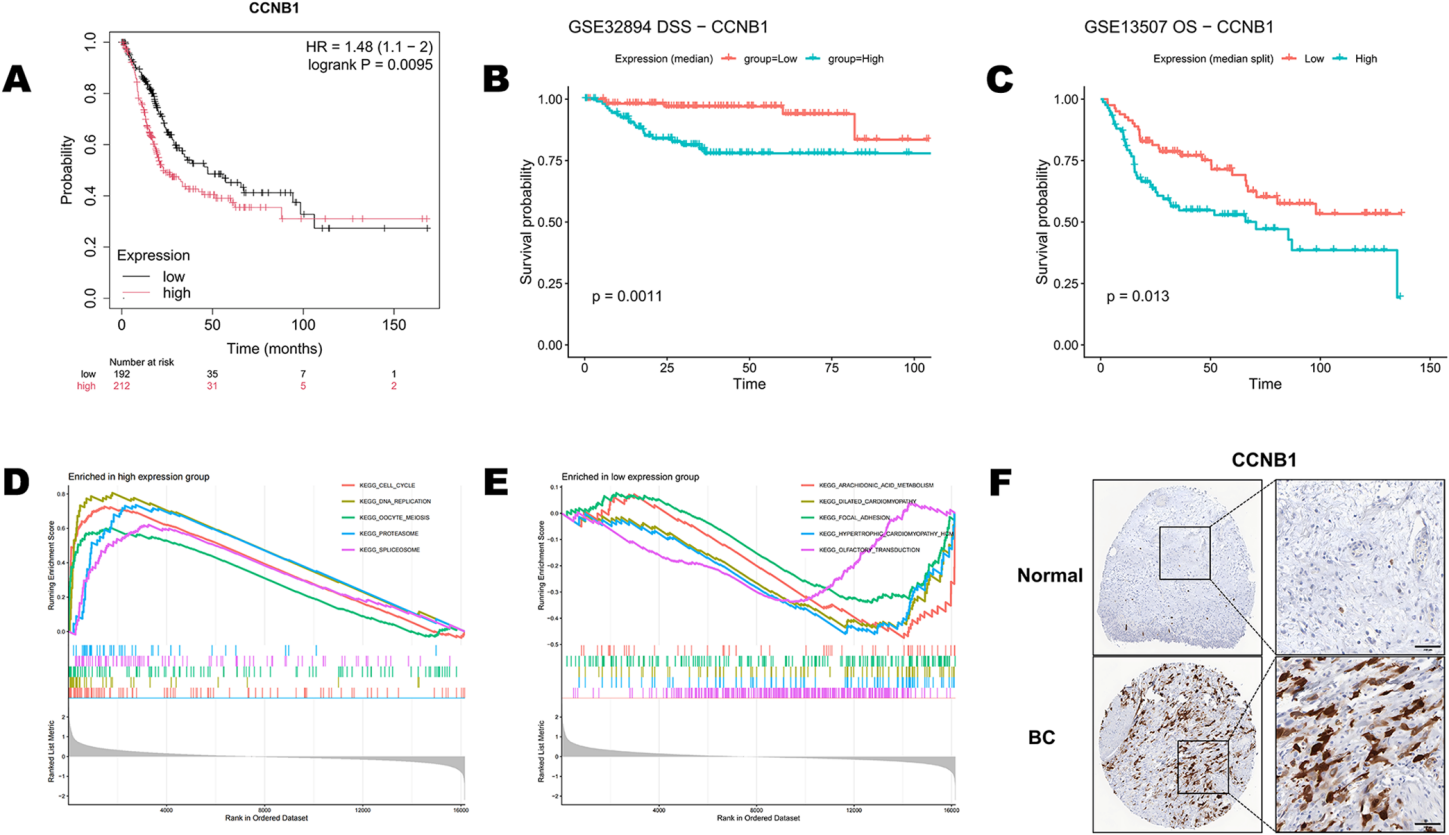
Prognostic significance of CCNB1 and validation of associated pathways and protein expression. (A) Kaplan–Meier overall survival (OS) analysis in the TCGA bladder cancer cohort stratified by median CCNB1 expression (high vs low). Hazard ratio (HR) and log-rank P value are shown. (B) Kaplan–Meier disease-specific survival (DSS) analysis in the GEO cohort GSE32894 stratified by median CCNB1 expression. (C) Kaplan–Meier overall survival (OS) analysis in the GEO cohort GSE13507 stratified by median CCNB1 expression. (D, E) Gene set enrichment analysis (GSEA) comparing CCNB1-high and CCNB1-low tumors: (D) enrichment of mitosis/cell-cycle-related pathways in the CCNB1-high group; (E) enrichment of immune-regulatory and metabolism-related pathways in the CCNB1-low group. (F) Immunohistochemistry (IHC) of CCNB1 in normal bladder tissue and bladder cancer tissue showing stronger nuclear/cytoplasmic staining in tumors (left, low magnification; right, corresponding higher magnification).

To explore the potential biological basis, we performed GSEA to compare tumors with high versus low CCNB1 expression. Tumors with high CCNB1 expression were significantly enriched for mitosis/cell-cycle-related pathways, including the G2/M checkpoint, E2F targets, and chromosome segregation (Figure 6D), whereas the low CCNB1 expression group showed enrichment of immune-regulatory and metabolism-related pathways, such as cytokine signaling and xenobiotic metabolism (Figure 6E). Moreover, GSEA results for the remaining hub genes (AURKB, CA2, CCNA2, CCNB2, and TK1) also showed enrichment signals related to cell-cycle-associated processes (Figure S3A,B).

Immunohistochemistry (IHC) further demonstrated markedly stronger CCNB1 staining in tumor tissues compared with normal tissues with prominent nuclear/cytoplasmic positivity in tumors and weak to nearly negative staining in normal tissues (Figure 6F). In addition, the other hub genes generally exhibited immunoreactivity in tumor tissues stronger than that in normal tissues at the protein level (Figure S4). Collectively, CCNB1 shows a robust association with poor prognosis across multiple cohorts, supported at the protein level, indicating its potential utility for prognostic evaluation and risk stratification in BC.

### Molecular Docking and Dynamics Simulations Confirm Stable Binding of Swertiamarin to the CCNB1 Active Site

Using multiple platforms to predict targets of GR active constituents, we found that CCNB1 exhibited strongly predicted binding to several compounds. Compound prioritization was guided by a CCNB1-centered, target-relevant ranking rather than literature prevalence alone. Integration of docking results for all gene–compound combinations (Figure 7A and Table S2) showed that CCNB1 exhibited relatively low binding energies with multiple small molecules (approximately −9.8 to −7.0 kcal/mol), indicating strong predicted binding affinities. Among these, the CCNB1–swertiamarin complex displayed the lowest binding energy and the most favorable energetic profile and was therefore selected for further investigation. Docking conformation analysis indicated that swertiamarin can stably occupy the active pocket of CCNB1, forming hydrogen bonds with key residues, such as GLU-18 and HIS-174, together with multiple hydrophobic interactions that collectively stabilize the complex (Figure 7B). The RMSD curves showed slight fluctuations at the beginning of the simulation, followed by gradual convergence to a stable plateau at approximately 40 ns (Figure 7C), indicating that the complex reached an equilibrium conformation and remained stable thereafter. RMSF analysis revealed that most residues displayed only minor fluctuations (Figure 7D), suggesting that ligand binding did not induce major conformational changes in the protein backbone. The radius of gyration (*R*_g_) remained essentially stable throughout the simulation (Figure 7E), indicating a relatively compact global conformation of the protein. The solvent-accessible surface area (SASA) also exhibited only slight fluctuations (Figure 7F), further suggesting that no marked folding or unfolding occurred in the protein–ligand system during the simulation. The Gibbs free energy landscape constructed from principal component analysis (Figure 7G,H) showed multiple low-energy basins for the CCNB1–swertiamarin complex, with the lowest-energy conformation corresponding to a particularly stable binding state. Taken together, the molecular docking and MD simulations consistently indicate, from both structural and energetic perspectives, that CCNB1 engages in a stable and favorable binding interaction with swertiamarin, providing a theoretical basis for CCNB1 as a potential therapeutic target in BC and for swertiamarin as a candidate small-molecule inhibitor.

**Figure 7:**
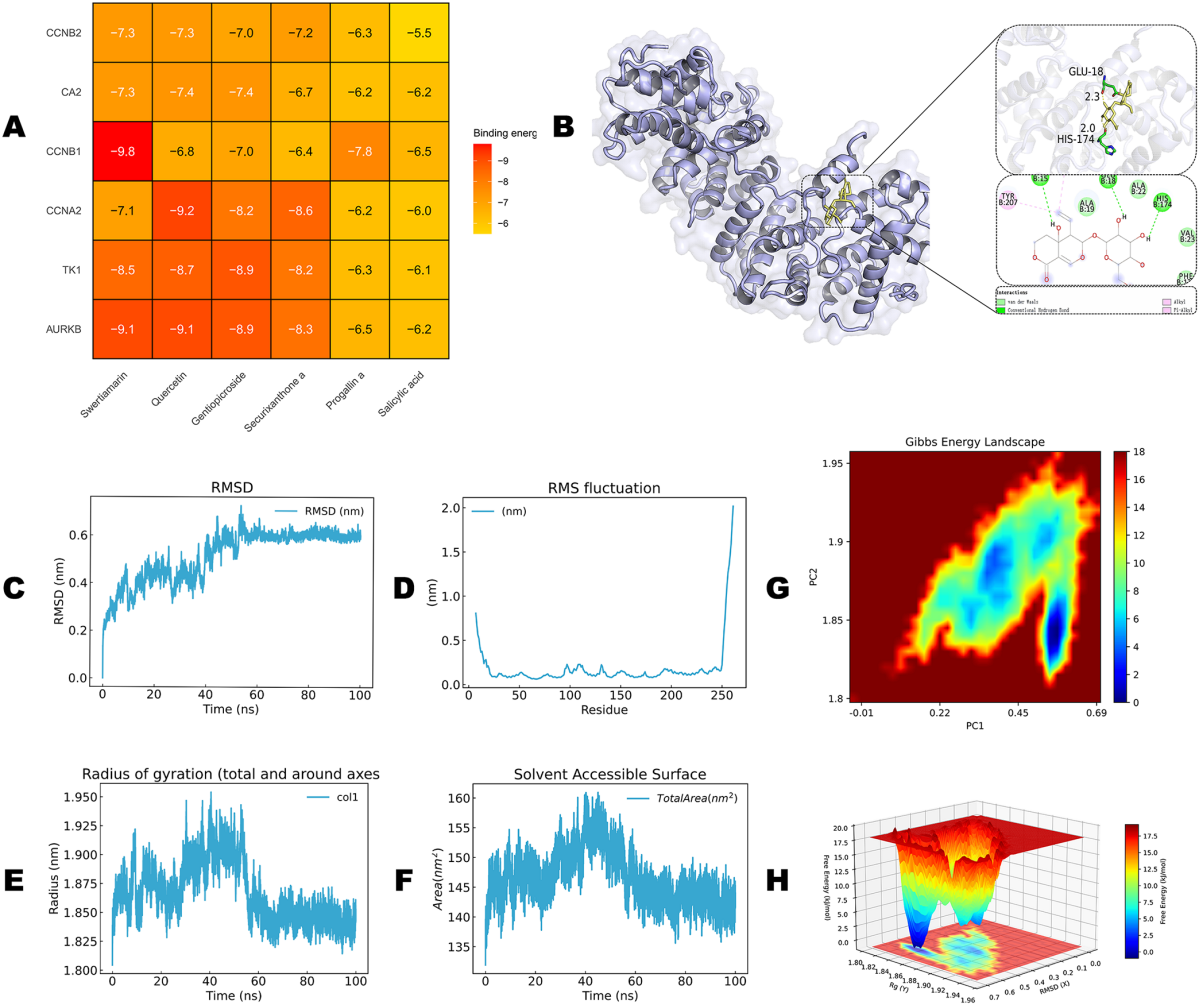
Molecular docking and dynamics simulation analysis of Swertiamarin with CCNB1. (A) Heatmap of binding energies (kcal/mol) for the core medicinal component of GR. (B) Representative binding pose of Swertiamarin within the CCNB1 binding pocket. (C) RMSD of the protein backbone versus simulation time (ns) during the 100 ns molecular dynamics simulation. (D) RMSF per residue of CCNB1 during the simulation. (E) *R*_g_ of CCNB1 versus time, indicating protein compactness. (F) SASA of the protein versus time. (G) Gibbs free energy landscape projected onto the first two principal components (PC1, PC2) from trajectory principal component analysis (PCA). Lower-energy regions denote more stable conformations. (H) Gibbs free energy landscape as a function of *R*_g_ and RMSD, illustrating complex conformational stability.

## Discussion

In this study, we integrated multicohort transcriptomic data, network pharmacology, machine learning, and computer-aided molecular simulations to systematically identify potential targets and mechanisms of action of the active constituents of GR in bladder cancer. We identified six robust hub genes (CA2, AURKB, CCNB1, CCNB2, CCNA2, and TK1) that were consistently upregulated and formed the basis of a high-accuracy artificial neural-network diagnostic model. Among these, CCNB1 was closely associated with cell-cycle activation and adverse clinical outcomes. Molecular docking and molecular dynamics simulations demonstrated a stable and energetically favorable interaction between swertiamarin and CCNB1, supporting CCNB1 as a druggable target and providing a mechanistic basis for the therapeutic effects of GR in bladder cancer.

Bladder cancer is characterized by marked heterogeneity, a high recurrence rate, and strong treatment resistance. Despite advances in surgery, chemotherapy, immunotherapy, and targeted therapy, overall therapeutic efficacy remains constrained by multiple factors.^19–21^ Natural products have emerged as an important potential source of therapeutics owing to their multitarget properties, low toxicity, and capacity to modulate complex biological networks,^22^ yet their systemic mechanisms of action remain incompletely understood.^23^ By leveraging systems biology, network pharmacology, machine learning, and molecular simulations, we established a multilevel evidence chain linking “herbal medicine–component–target–pathway–phenotype” and systematically dissected the antibladder cancer potential of GR and its core constituent swertiamarin.

By integrating TCGA, GEO, disease databases, WGCNA modules, and target prediction, we obtained eight cross-cohort–consistent candidate genes, thereby reducing bias arising from any single data source. By further combining three machine-learning algorithms, we screened six hub genes and constructed a neural-network diagnostic model that achieved diagnostic performance superior to that of conventional models. Compared with previous studies that largely relied on a single modeling approach, our multialgorithm cross-screening strategy enhanced the robustness of feature-gene selection and its potential for clinical translation. The six-gene diagnostic model developed here may in the future be applied to preoperative risk stratification and recurrence surveillance in bladder cancer, providing a molecular tool for precision diagnosis and treatment. Consistent with the literature, we observed widespread upregulation of genes involved in cell-cycle regulation in both the TCGA and GEO cohorts. This observation aligns with established molecular hallmarks of bladder cancer—dysregulation of the G2/M checkpoint, aberrant chromosome segregation, and rapid proliferative division^24,25^ and with the roles of CCNB1, CCNA2, AURKB, TK1, and related genes as proliferation drivers in bladder cancer and multiple solid tumors.^26–28^ Together, these findings underscore the reliability of our disease-level results.

Among these hub genes, the expression pattern and prognostic value of CCNB1 were particularly prominent. Multicohort survival analyses showed that high expression of CCNB1 was significantly associated with poorer overall survival. GSEA revealed that high CCNB1 expression was enriched in proliferative pathways, including the cell cycle, G2/M checkpoint, mitotic spindle assembly, and activation of the E2F target genes. In contrast, low expression of CCNB1 was significantly associated with immune-related processes, such as complement activation, antigen presentation, and cytokine signaling. This “enhanced proliferation–suppressed immunity” pattern provides a plausible biological explanation for why CCNB1 acts as an adverse prognostic factors.^29,30^ IHC further confirmed marked upregulation of CCNB1 in bladder cancer tissues, reinforcing its reliability as a diagnostic and prognostic biomarker.

Using multiple platforms to predict targets of GR active constituents, we found that CCNB1 exhibited strong predicted binding to several compounds. Among these, swertiamarin showed the lowest binding energy; molecular docking indicated that it can stably occupy the active pocket of CCNB1 and form hydrogen bonds and hydrophobic interactions with key residues. Molecular dynamics simulations further confirmed the high conformational stability of the complex, providing structural evidence for a strong swertiamarin–CCNB1 interaction. Functionally, CCNB1 drives cells into mitosis by forming a complex with CDK1.^31,32^ We therefore speculate that swertiamarin may competitively or allosterically interfere with CCNB1 function, thereby inhibiting cell-cycle progression at the molecular level. This hypothesis offers a rational molecular explanation for the antitumor effects of GR and lays the groundwork for its further development as a natural targeted therapeutic agent.

The translational potential of swertiamarin is also dependent on exposure at the tumor site. Published pharmacokinetic studies in rodents suggest that swertiamarin is rapidly absorbed after oral dosing but exhibits low absolute oral bioavailability (approximately 5.6–7.6%) with a short half-life of about 1 hour.^33,34^ Other reports have similarly described low absolute bioavailability (∼10.3%) in rats.^35^ These properties may limit the systemic efficacy without optimization. Notably, bladder cancer offers practical opportunities for local drug delivery (e.g., intravesical instillation), and formulation approaches or lead-optimization strategies may further improve the exposure. Future work should integrate PK/PD^36,37^ profiling and optimized delivery to validate whether swertiamarin can achieve therapeutically relevant concentrations in vivo.

This study has several limitations, including heterogeneity in publicly available data, the lack of prospective clinical validation of the diagnostic model, the need for experimental confirmation of the simulation results, and uncertainty as to whether swertiamarin directly regulates the CCNB1/cell-cycle axis in vivo. Future work will focus on: developing qPCR- or IHC-based CA2/CCNB1 diagnostic panels using clinical specimens; systematically validating, in vitro and in vivo, the regulatory effects of swertiamarin on the bladder cancer cell cycle, DNA damage response, and immune microenvironment; performing medicinal chemistry optimization using swertiamarin as a lead structure; and elucidating the roles of CA2 and CCNB1 in metabolic reprogramming and immune regulation using single-cell and spatial omics.

## Conclusions

In summary, from the perspectives of systems biology and network pharmacology, this study constructed a multilevel evidence chain for the action of GR in bladder cancer, identified CCNB1 as a key target with dual diagnostic and prognostic value, and elucidated the potential molecular basis of the stable interaction between swertiamarin and CCNB1. These findings provide new strategies and evidence for molecular diagnosis and natural-compound-based targeted therapy in bladder cancer.

## Methods

### Data Preprocessing and Construction of the Weighted Gene Coexpression Network

Bulk RNA-seq count data for bladder cancer (BC) and matched normal tissues were obtained from TCGA-BLCA. Differential expression analysis was conducted using the DESeq2 package in R, which fits a negative binomial generalized linear model and applies a Benjamini–Hochberg false discovery rate (FDR) correction for multiple testing. Genes with adjusted *P* (FDR) < 0.05 and |log2FC| > 1 were defined as TCGA-derived DEGs. For GEO microarray data sets (GSE13507 and GSE40355),^38^ the two data sets were preprocessed and merged, and batch effects were removed prior to downstream analyses. Differential expression analysis was then performed using the limma framework (linear model with empirical Bayes moderation), with multiple testing adjusted by Benjamini–Hochberg FDR. Genes with adjusted *P* (FDR) < 0.05 and |log2FC| > 0.585 were defined as GEO-derived DEGs.

A weighted gene coexpression network was constructed from the GEO-derived expression matrix using weighted gene coexpression network analysis (WGCNA). After normalization with limma, a topological overlap matrix (TOM) was generated with β = softPower. Coexpression modules were identified and merged by dynamic tree cutting (minModuleSize = 60, cutHeight = 0.25). Module–trait correlations were assessed using Pearson’s correlation, with *P* < 0.05 considered statistically significant.

Bladder cancer-related genes (disease-associated genes, DAGs) were retrieved from the Comparative Toxicogenomics Database­(CTD),^39^ Online Mendelian Inheritance in Man (OMIM),^40^ and GeneCards databases.^41^ In CTD, the disease term “Urinary Bladder Neoplasms” was used to obtain disease–gene associations, and the export was restricted to *Homo sapiens*. In GeneCards, genes were searched using the keyword “bladder cancer”, and candidates with a relevance score ≥ 1.0 were retained. In OMIM, disease-gene records related to bladder cancer were collected, and genes were filtered by Approved Symbol (HGNC-approved gene symbols) to ensure standardized gene identifiers. After removing duplicates and unifying gene symbols across databases, the retrieved DAG sets were compared, and the overlapping genes were used for downstream analyses. All databases were queried and accessed up to October 2025.

### Target Prediction for GR

Based on Lipinski’s rule of five (molecular weight ≤ 500, Log*P* ≤ 5, hydrogen bond donors ≤ 5, hydrogen bond acceptors ≤ 10), active herbal components of GR were extracted from the HERB 2.0 ^42^ and SymMap^43^ databases. Target genes for each key active compound (drug–target genes, DTGs) were predicted using SwissTargetPrediction^44^ (probability > 0.7), SEA,^45^ and the ChEMBL platform.^46^

### Identification of Disease–Drug Intersecting Genes and Enrichment Analysis

Common genes shared across five gene sets (TCGA-DEGs, GEO–DEGs, WGCNA module genes, DAGs, and DTGs) were extracted and regarded as candidate biomarkers. These shared genes were subjected to multilevel functional annotation: Gene Ontology (GO) enrichment analysis to characterize biological process (BP), molecular function (MF), and cellular component (CC); Kyoto Encyclopedia of Genes and Genomes (KEGG) pathway enrichment analysis to identify signaling pathways; and Disease Ontology (DO) enrichment analysis to explore disease associations.

### Identification of Key Genes Based on Machine Learning

Gene selection was performed using LASSO (10-fold CV; λ_min = 0.002809829, λ_1se = 0.07291573; features retained at λ_min), SVM-RFE (10-fold CV; kernel = linear; C = 10), and random forest (ntree = 500 initially; 176 trees in the final model; feature sampling ratio mtry/p = 2/8 = 0.25), and overlapping genes were defined as hub genes (HGs). The HGs were imported into the GeneMANIA^47^ online platform to construct a protein–protein interaction (PPI) network.

### Construction of an Artificial Neural-Network Model and Prognostic Evaluation

An artificial neural-network model based on the hub genes was constructed, and its performance was evaluated by calculating the area under the receiver operating characteristic curve (AUC) in the GEO training set. To assess the generalizability of the model in an independent data set, the TCGA cohort was randomly divided into training and testing sets at a 7:3 ratio. Model sensitivity and specificity were then evaluated in the TCGA testing set (30% of samples). For the six hub genes, gene set enrichment analysis (GSEA) based on genome-wide ranked lists was performed using MSigDB Hallmark and KEGG gene sets, with a false discovery rate (FDR) < 0.25. Survival analyses for the HGs were conducted using the Kaplan–Meier Plotter^48^ online tool, and hazard ratios (HRs) with 95% confidence intervals (CIs) were reported. Immunohistochemical staining images of the hub genes in BC and normal bladder tissues were obtained from the Human Protein Atlas.^49^

### Molecular Docking and Molecular Dynamics Simulation

Canonical SMILES strings and three-dimensional (3D) structures of the core active compounds were retrieved from the PubChem database.^50^ Protein structures corresponding to key target genes were obtained from the UniProt database,^51^ and molecular docking was performed using the CB-Dock2 ^52^ online platform. The optimal docking complexes, defined as those with the lowest predicted binding free energy (Δ*G*), were subjected to GROMACS simulations. Molecular dynamics simulations of 100 ns were carried out in the NPT ensemble by using the CHARMM36 force field (TIP3P water model, 150 mM ionic strength, 310 K, 1 bar). System stability was evaluated using the root-mean-square deviation (RMSD), root-mean-square fluctuation (RMSF), and radius of gyration (*R*_g_).

## Supplementary Material



## Notes

Identification of differentially expressed genes and WGCNA module selection; Kaplan–Meier survival analyses of five genes; GSEA results; immunohistochemical staining images; summary of sample sizes and DEGs; docking binding free energies between key active components and key proteins; and database names and website URLs (PDF)
