# Supplementary material for: Integrative Transcriptomics, Machine Learning, and Molecular Dynamics Reveal Honghua Longdan (Gentiana rhodantha)‑Modulated Therapeutic Targets in Bladder Cancer
Source: ACS Omega. 2026 Mar 6;11(11):17996–8009. doi: 10.1021/acsomega.5c12612 (PMC13019180; doi:10.1021/acsomega.5c12612)
Supplement: Supplementary file 1 [file ao5c12612_si_001.pdf]

Integrative Transcriptomics, Machine Learning, and Molecular Dynamics Reveal Honghua Longdan (*Gentiana rhodantha*)–Modulated Therapeutic Targets in Bladder Cancer

Qinsha Wang[a,#], Haihong Wang[a,#],Peng Lan[b], Bing Yang [a], Jia Deng[a], Kangmin Zhou[a], Dongxin Tang[a,*]

[a]*Guizhou University of Traditional Chinese Medicine,50 Shidong Road, Nanming District, 550002, Guiyang, Guizhou Province, China.*

[b]*Department of Emergency Medicine, The Traditional Chinese Medicine Hospital of Longquanyi, 222Qingtaishan Road, Damian Subdistrict, Chengdu, 610100, Sichuan Province, China*

[#]*Equal contribution*

Email: Heminganking@sina.com

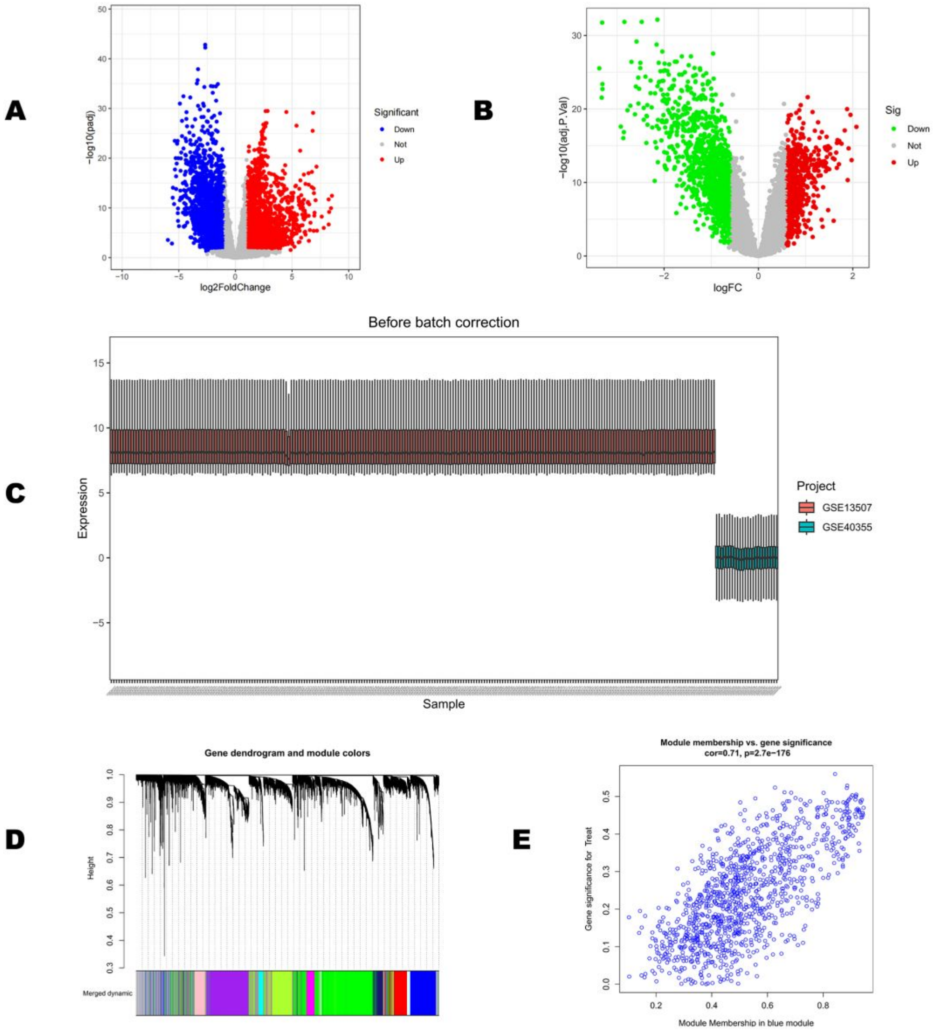

Figure S1. Identification of differentially expressed genes and selection of the key WGCNA module associated with bladder cancer. (A) Volcano plot of differential expression between bladder cancer and normal tissues in the TCGA-BLCA cohort. The x-axis indicates log2 fold change and the y-axis indicates −log10 (adjusted P value). Red dots represent upregulated genes, blue dots represent downregulated genes, and grey dots indicate non-significant genes. (B) Volcano plot of differential expression between tumor and normal samples in the GEO cohort (GSE13507 and GSE40355). The x-axis indicates logFC and the y-axis indicates −log10 (adjusted P value). Red dots represent upregulated genes, green dots represent downregulated genes, and grey dots indicate non-significant genes. (C) Boxplots of expression values for GSE13507 and GSE40355 before batch correction, showing systematic distribution differences between datasets and indicating pronounced batch effects. (D) Gene clustering dendrogram and corresponding module color assignment from WGCNA. (E) DF plot for the module most strongly associated with the disease trait: scatter plot of module membership (MM) versus gene significance (GS) for genes in

this module, used to evaluate the relationship between intramodular connectivity and disease relevance.

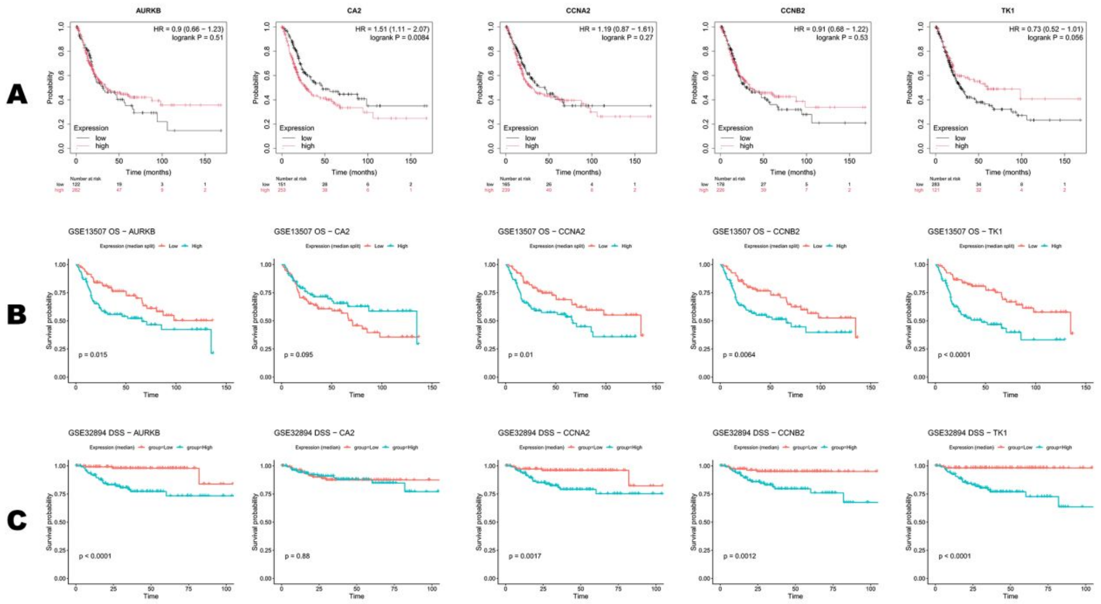

Figure S2. Prognostic analysis of five genes in bladder cancer. (A) Kaplan-Meier (KM) survival curves for AURKB, CA2, CCNA2, CCNB2, and TK1 genes from the KM database. Each gene is divided into high expression and low expression groups, with the corresponding hazard ratio (HR) and P values indicated for each gene. (B) KM curves from the GSE13507 dataset, showing the survival analysis results for AURKB, CA2, CCNA2, CCNB2, and TK1 genes in this dataset. (C) KM curves from the GSE32894 dataset, showing the survival analysis results for AURKB, CA2, CCNA2, CCNB2, and TK1 genes in this dataset. The curves represent high expression and low expression groups, demonstrating the association between gene expression and patient survival.

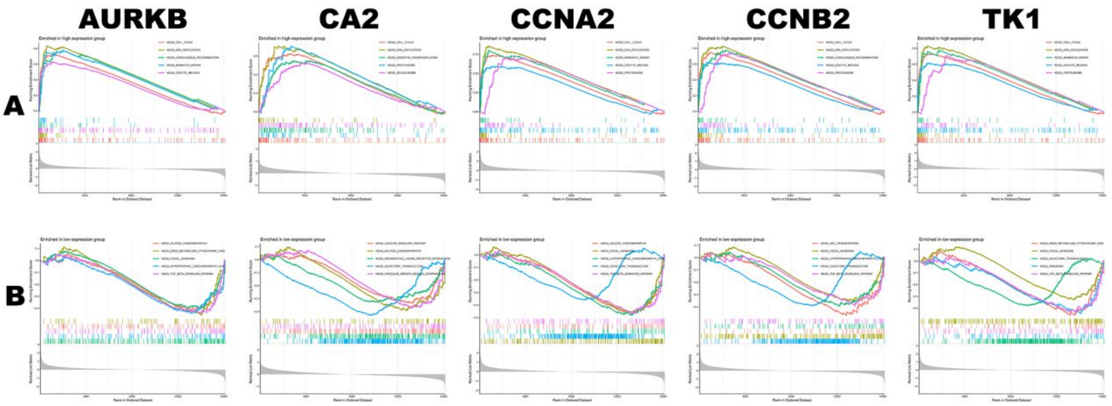

Figure S3. Gene set enrichment analysis (GSEA) results. (A) GSEA enrichment curves for AURKB, CA2, CCNA2, CCNB2, and TK1 in the high expression group. The y-axis represents the normalized enrichment score (NES), and the x-axis shows the sample ranking. Different color curves represent different clinical groups, with the threshold lines indicating significance. (B) GSEA enrichment curves for the same genes in the low expression group, comparing enrichment trends across different groups, with changes in gene enrichment levels across sample groups shown.

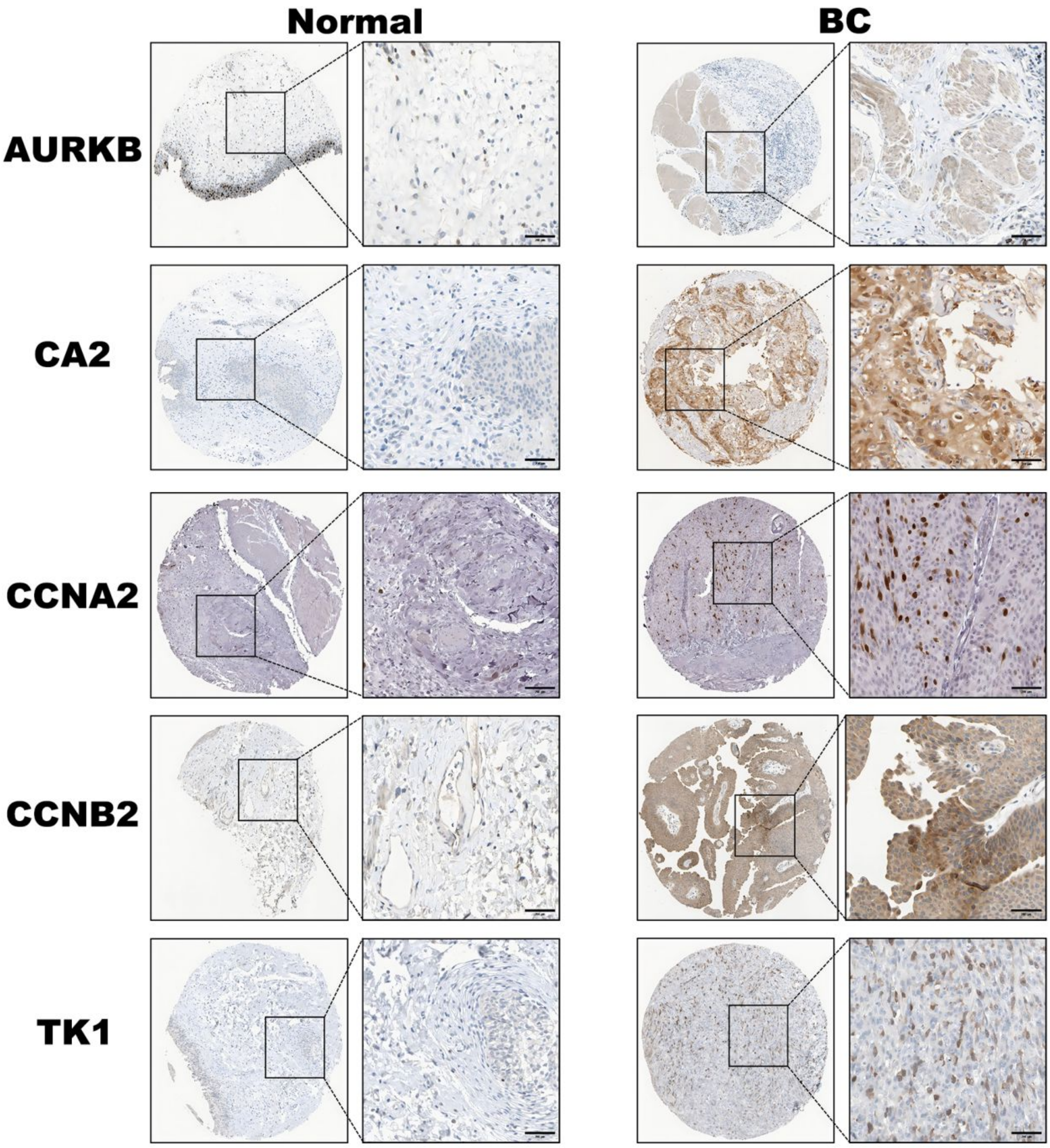

Figure S4. Immunohistochemical staining of AURKB, CA2, CCNA2, CCNB2, and TK1 in normal and bladder cancer tissues. Immunohistochemical staining showing the expression of AURKB, CA2, CCNA2, CCNB2, and TK1 in normal bladder tissue and bladder cancer tissue. The left side of each gene shows expression in normal tissue, while the right side shows expression in bladder cancer tissue.

Table S1. Summary of sample sizes and DEGs in TCGA-BLCA and merged GEO cohorts.

<table>
<tr><th>Cohort</th><th>Dataset</th><th>Group</th><th>N</th><th>DEG thresholds</th><th>DEG total</th><th>Up</th><th>Down</th></tr>
<tr><td>TCGA-BLCA</td><td>TCGA</td><td>Normal</td><td>19</td><td>adj.P<0.05; |log2FC|>1</td><td>4704</td><td>2700</td><td>2004</td></tr>
<tr><td>TCGA-BLCA</td><td>TCGA</td><td>Tumor</td><td>412</td><td>adj.P<0.05; |log2FC|>1</td><td>4704</td><td>2700</td><td>2004</td></tr>
<tr><td>Merged GEO</td><td>GSE13507</td><td>Control</td><td>67</td><td>adj.P<0.05; |logFC|>0.585</td><td>1962</td><td>641</td><td>1321</td></tr>
<tr><td>Merged GEO</td><td>GSE13507</td><td>Treat</td><td>165</td><td>adj.P<0.05; |logFC|>0.585</td><td>1962</td><td>641</td><td>1321</td></tr>
<tr><td>Merged GEO</td><td>GSE40355</td><td>Control</td><td>8</td><td>adj.P<0.05; |logFC|>0.585</td><td>1962</td><td>641</td><td>1321</td></tr>
<tr><td>Merged GEO</td><td>GSE40355</td><td>Treat</td><td>16</td><td>adj.P<0.05; |logFC|>0.585</td><td>1962</td><td>641</td><td>1321</td></tr>
<tr><td>Merged GEO</td><td>Merged (GSE13507+GSE40355)</td><td>Control</td><td>75</td><td>adj.P<0.05; |logFC|>0.585</td><td>1962</td><td>641</td><td>1321</td></tr>
<tr><td>Merged GEO</td><td>Merged (GSE13507+GSE40355)</td><td>Treat</td><td>181</td><td>adj.P<0.05; |logFC|>0.585</td><td>1962</td><td>641</td><td>1321</td></tr>
</table>

Note: DEGs were defined as differentially expressed genes. TCGA-DEGs were identified using adjusted P<0.05 and |log2FC|>1; GEO-DEGs were identified using adjusted P<0.05 and |logFC|>0.585 (fold change >1.5).

Table S2. Docking binding free energies (kcal/mol) between key active components of Gentiana rhodantha and key proteins

	**Gentiopicroside**	**Progallin a**	**Quercetin**	**Salicylic acid**	**Securixanthone a**	**Swertiamarin**
CCNB2	-7.0	-6.3	-7.3	-5.5	-7.2	-7.3
CA2	-7.4	-6.2	-7.4	-6.2	-6.7	-7.3
CCNA2	-8.2	-6.2	-9.2	-6.0	-8.6	-7.1
CCNB1	-7.0	-7.8	-6.8	-6.5	-6.4	-9.8
AURKB	-8.9	-6.5	-9.1	-6.2	-8.3	-9.1
TK1	-8.9	-6.3	-8.7	-6.1	-8.2	-8.5

Table S3. Database Names and Website URLs

**Database Name**	**Website URL**
The Cancer Genome Atlas Program (TCGA)	https://www.cancer.gov/ccg/research/genome-sequencing/tcga
GEO （GENE EXPRESSION OMNIBUS）	https://www.ncbi.nlm.nih.gov/geo/
GeneCards: The Human Gene Database	https://www.genecards.org/
OMIM	https://www.omim.org/
CTD(Comparative Toxicogenomics Database)	https://ctdbase.org/
SymMap	http://www.symmap.org/
HERB2.0	http://47.92.70.12/Detail/?v=HERB002280&label=Herb

Database Name	Website URL
SwissTargetPrediction	https://www.swisstargetprediction.ch/
ChEMBL	https://www.ebi.ac.uk/chembl/
SEA	https://sea.bkslab.org/
GENEMNIA	https://genemania.org/
CB-Dock2	https://cadd.labshare.cn/cb-dock2/index.php
